# (Epi)genetic control of human trophoblast invasion

**DOI:** 10.3389/fgene.2014.00038

**Published:** 2014-02-17

**Authors:** Marie van Dijk, Cees Oudejans

**Affiliations:** ^1^Department of Clinical Chemistry, VU University Medical CenterAmsterdam, Netherlands; ^2^Institute for Cardiovascular Research, VU University Medical CenterAmsterdam, Netherlands

**Keywords:** trophoblast, placenta, differentiation, methylation, invasion, microRNAs, Wnt signaling, placental diseases

During pregnancy the placenta is essential for the growing fetus as it provides oxygen and nutrients and accomplishes waste disposal by connecting the maternal and fetal circulations. To form a functional placenta the fetomaternal interface must be properly developed. For this, fetal extravillous trophoblasts need to invade the maternal placental bed thereby remodeling the maternal spiral arteries from low-capacity high-resistance to high-capacity low-resistance vessels. Inadequate trophoblast invasion can be found at the origin of a variety of pregnancy complications and diseases, such as IUGR (Intra Uterine Growth Restriction), familial early-onset pre-eclampsia, and the HELLP syndrome (Hemolysis, Elevated Liver Enzymes, Low Platelets). This research topic will discuss the various genetic and epigenetic aspects that so far have been identified to control this biological process of trophoblast invasion.

The first level of control on invasion is control on trophoblast differentiation. Trophoblasts differentiate from villous cytotrophoblasts to proliferating column extravillous trophoblasts and finally to non-proliferating invasive extravillous trophoblasts. Important players in this differentiation cascade are Wnt signaling components (Knöfler and Pollheimer, 2013), microRNAs (Doridot et al., 2013) and DNA methylation (Novakovic and Saffery, 2013) and its regulators, i.e., DNA methyltransferases and TET proteins (Logan et al., 2013).

Knöfler and Pollheimer (2013) describe how the Wnt signaling pathway contributes to differentiation of extravillous trophoblasts and their invasive properties is indicated by the translocation of β-catenin to the nucleus and the upregulation of TCF-4. Canonical Wnt activity has been shown to promote trophoblast invasion, where hyperactivation leads to trophoblast disorders such as choriocarcinoma and hydatidiform moles, while downregulation of the Wnt signaling cascade can be found in pre-eclampsia.

MicroRNAs (miRNAs) are able to affect gene expression by modulating mRNA stability and/or translation. Doridot et al. (2013) describe the role of miRNAs in regulating various aspects of placental development. Particular focus is on the effects of miRNAs on Notch signaling leading to aberrant trophoblast differentiation and invasion, and affecting angiogenesis at the fetomaternal interface. Secondly, differentiation of the villous cytotrophoblasts into syncytiotrophoblasts under miRNA control is discussed. Another important function of miRNAs involves modulation of the immunological balance of the uterine-embryo contact. Furthermore, two specific aspects are highlighted, i.e., miRNAs induced by hypoxia and miRNAs originating from imprinting clusters, as both play an important role in placental development.

DNA methyltransferases (DNMTs) and TET proteins, methylate and demethylate DNA, respectively, leading to changes in transcription. Logan et al. (2013) discuss their role in trophoblast differentiation from trophoblast stem cells to cytotrophoblasts to syncytiotrophoblasts and proliferating column trophoblasts. Partly under control of DNMTs and TETs the latter subsequently differentiate from an epithelial to a highly invasive mesenchymal phenotype.

The perspective by Novakovic and Saffery (2013) describes the extensive parallels that exist between trophoblast invasion and tumorigenesis. Not only molecular pathways are shared but also epigenetic mechanisms. The DNA methylation similarities between the placenta and cancer are discussed from global hypomethylation to placenta-specific tumor suppressor methylation to the occurrence of loss-of-imprinting. Furthermore, a common origin of epigenetic features in placenta and cancer is speculated from the simple explanation of the independent use of similar pathways or the more intriguing observation that placental mammals have a higher incidence of cancers indicating a reactivation of placental pathways in cancer.

Specific genes involved in aberrant trophoblast invasion have been identified via genome-wide linkage studies in families suffering from pregnancy-associated diseases like pre-eclampsia and the HELLP-syndrome as well as via candidate gene approaches (van Dijk and Oudejans, 2013). The potential functions of these genes in the etiology of disease are discussed including the role of epigenetic inheritance. Thulluru et al. (2013) describe a study on Nodal expression in the maternal decidua affecting, potentially via Activin-A upregulation, Nodal and STOX1 expression in extravillous trophoblasts. STOX1 was identified by genome-wide linkage analysis and inhibits trophoblast differentiation and invasion. Nodal is found to be upregulated in pre-eclamptic placentas and inhibits invasion of extravillous trophoblasts, while Activin-A is increased in serum of pre-eclamptic patients. As decidua-specific Nodal knockout mice show clear signs of poor placentation, i.e., IUGR and preterm birth, this study links multiple candidate genes involved in trophoblast invasion.

The contributions to this research topic clearly show the diversity of genetic and epigenetic components that individually play a role in the control of human trophoblast invasion in both healthy and diseased states of the placenta.
